# VSL#3 Resets Insulin Signaling and Protects against NASH and Atherosclerosis in a Model of Genetic Dyslipidemia and Intestinal Inflammation

**DOI:** 10.1371/journal.pone.0045425

**Published:** 2012-09-21

**Authors:** Andrea Mencarelli, Sabrina Cipriani, Barbara Renga, Angela Bruno, Claudio D'Amore, Eleonora Distrutti, Stefano Fiorucci

**Affiliations:** 1 Dipartimento di Medicina Clinica e Sperimentale, University of Perugia, Facoltà di Medicina e Chirurgia, Perugia, Italy; 2 Azienda Ospedaliera di Perugia, Perugia, Italy; Instutite of Agrochemistry and Food Technology, Spain

## Abstract

**Background:**

Signals generated by the inflammed intestine are thought to contribute to metabolic derangement. The intestinal microbiota contributes to instructing the immune system beyond the intestinal wall and its modulation is a potential target for treating systemic disorders.

**Aims:**

To investigate the pathogenetic role of low grade intestinal inflammation in the development of steatohepatitis and atherosclerosis in a model of genetic dyslipidemia and to test the therapeutic potential of a probiotics intervention in protecting against development of these disorders.

**Results:**

ApoE^−/−^ mice were randomized to receive vehicle or VSL#3, a mixture of eight probiotics, at the dose of 20×10^9^ colony-forming units/kg/day for three months alone or in combination with 0.2% of dextran sulfate sodium (DSS) in drinking water. Administering DSS to ApoE^−/−^ mice failed to induce signs and symptoms of colitis but increased intestinal permeability to dextran FITC and, while had no effect on serum lipids, increased the blood levels of markers of liver injury and insulin resistance. DSS administration associated with low level inflammation of intestinal and mesenteric adipose tissues, caused liver histopathology features of steatohepatitis and severe atherosclerotic lesions in the aorta. These changes were prevented by VSL#3 intervention. Specifically, VSL#3 reversed insulin resistance, prevented development of histologic features of mesenteric adipose tissue inflammation, steatohepatitis and reduced the extent of aortic plaques. Conditioned media obtained from cultured probiotics caused the direct transactivation of peroxisome proliferator-activated receptor-γ, Farnesoid-X-receptors and vitamin D receptor.

**Conclusions:**

Low grade intestinal inflammation drives a transition from steatosis to steatohepatitis and worsens the severity of atherosclerosis in a genetic model of dyslipidemia. VSL#3 intervention modulates the expression of nuclear receptors, corrects for insulin resistance in liver and adipose tissues and protects against development of steatohepatitis and atherosclerosis.

## Introduction

Metabolic syndrome is a diagnostic category, based on a cluster of risk factors (hyperglycemia/diabetes, abdominal obesity, hypertriglyceridemia, low HDL cholesterol and hypertension), which identifies subjects at high risk for development of type 2 diabetes mellitus and cardiovascular diseases [1]. The metabolic syndrome is also a strong predictor for nonalcoholic fatty liver disease (NAFLD) a condition ranging from simple lipid accumulation in the liver (steatosis) to steatosis combined with inflammation (steatohepatitis/NASH) [2], [3]. Subjects with the metabolic syndrome have a 4-fold increase in hepatic fatty content [4], and the presence of the metabolic syndrome among NAFLD patients increases the likelihood for advanced liver disease [5]–[7]. Compared with the general population cardiovascular mortality is increased by two folds among NASH patients [8]–[10]. Similar to insulin resistance, increased levels of fibrinogen, C-reactive protein (CRP) and plasminogen activator inhibitor-1 (PAI-1), liver fat accumulation is an independent risk factor for development of cardiovascular diseases [11]–[13].

Although hepatic steatosis is generally asymptomatic, relatively benign and reversible, the transition from steatosis to steatohepatitis represents a critical step in the progression to more severe forms of liver damage culminating in hepatic fibrosis and cirrhosis [14]. At present, the actual risk factors that drive hepatic inflammation during the progression from steatosis to steatohepatitis are largely unknown. There is a growing body of evidence suggesting that the progression of NAFLD to NASH depends on interactions between genetic and environmental factors [15]. The latter include small intestine bacterial overgrowth and bacterial translocation through the intestinal wall [16]–[19]. Gut bacteria might contribute to the pathogenesis of liver inflammation by multiple mechanisms, including increasing gut luminal ethanol production [20], metabolizing dietary choline (required for very-low-density lipoprotein synthesis and hepatic lipid export) [21] and/or by releasing lipopolysaccharide [22] which is likely to activate local and systemic proinflammatory pathways [23]–[26]. Recently, it has been reported that probiotics and prebiotics might be beneficial in reducing hepatic steatosis induced by a high-fat diet providing further experimental evidence for a role of gut-derived antigens in the onset of the NASH [27], [28].

Apoliprotein (Apo) E is a ligand found in remnant lipoproteins that is recognized by various receptors in the liver. In humans, ApoE deficiency, or the presence of mutant forms of ApoE, results in type III hyperlipidemia characterized by the presence of elevated VLDL lipoproteins and early age onset of atherosclerosis. ApoE deficient mice (ApoE^−/−^) are a widely used model of atherosclerosis, hyperlipidemia and steatosis [29]. Thus, while ApoE^−/−^ mice develop a severe hyperlipidemia and atherosclerosis on a standard diet, they fail to develop liver inflammation (NASH-like lesions), unless exposed to an additional hitting agent, making this setting a suitable model for testing the effects of therapeutic intervention on progression of lipid-related disorders in the liver and cardiovascular system. In the present study we have investigated the effects of VSL#3, a mixture of eight probiotic strains, in the progression of liver and vascular damage caused by challenging ApoE^–/–^ with a low concentration of dextrane sulphate sodium (DSS), a well characterized intestinal barrier braking agent.

**Table 1:** Effects of VSL#3 intervention on clinical and biochemical parameters.

Biomarkers	Wild type naive	ApoE^−/−^ naive	Apoe^−/−^ + VSL#3	ApoE^−/−^ + DSS	ApoE^−/−^ + DSS + VSL#3
**Initial body weight (g)**	33.5±1.5	31.3±0.5	31.9±0.5	31.4±0.7	31.6±0.6
**Final body weight (g)**	34.3±1.9	31.4±0.5	32.4±0.6	32.80±0.7	32.30±0.4
**Food intake**	4.2±0.7	4.2±0.1	4.3±0.2	4.2±0.2	4.4±0.3
**Total cholesterol (mg/dl)**	62.7±9.6	432.80±0.7 *	414±40.3 *	480±45.0 *	430.3±63.7 *
**LDLc (mg/dl)**	5.3±0.7	295.5±39.7 *	299.9±34.3 *	375.0±40.1 *	319.0±52.7 *
**HDLc (mg/dl)**	51.3±9.4	86.1±5.7 *	99. 4±6.0 *	104.8±6 .2 *	100.7±11.2 *
**Triacylglycerols (mg/dl)**	29.0±1.0	70.0±10.0 *	56.7±6.5 *	57.6±4.3 *	51.0±5.4 *
**Glucose (mg/dl)**	138±8.3	150.5±14.9	140.6±12	153.2±11.3	156.8±7.5
**Insulin (µg/ml)**	0.8±0.2	7.8±0.8 *	5.8±1.3 *	9.3±0.7 *	5.5±1.0 #*
**AST (IU/l)**	42.7±2.3	38.2±11.7	29.3±5.4	89.2±36.3 * ^and^ **	29.0±3.3 #
**ALT (IU/l)**	68.6±3.3	69.38±7.1	66.3±18.4	119.3±25.5 * ^and^ **	64.5±10.4 #

The values are expressed as mean ± SE (n = 8–12). * p<0.05 ApoE^−/−^ experimental group versus naive wild type group; ** p<0.05 ApoE^−/−^ naïve group versus ApoE^−/−^ plus VSL#3 group; $ p<0.05 ApoE^−/−^ naive group versus ApoE^−/−^ plus DSS group; # p<0.05 ApoE^−/−^ plus DSS group versus ApoE^−/−^ plus DSS and VSL#3 group.

The results of these studies demonstrate that a low grade inflammation increases intestinal permeability and leads to insulin resistance, transition from steatosis to NASH and exacerbated atherosclerosis and that all these disorders are efficiently prevented by a therapeutic intervention with a probiotic preparation. The study establishes that intervention on the intestinal microbiota is an effective therapeutic option in the treatment of systemic disorders.

## Results

### Effects of VSL#3 intervention on clinical and biochemical parameters in ApoE^−/−^ mice

ApoE^−/−^ and wild-type mice (pair aged) had comparable body weight gain throughout the study (Table 1) and administration of 0.2% DSS to ApoE^−/−^ mice failed to induce alteration in the body weight and food intake (Table 1). Similarly, administration of the VSL#3 had no effect on these parameters (Table 1). Compared with naive wild-type, naive ApoE^−/−^ mice at 12 months of age had increased cholesterol and triacylglycerols plasma levels without changes in the plasma biomarkers of liver damage (Table 1, p<0.05; n = 8–12). In contrast, exposure to DSS resulted in a robust increase in ALT and AST plasma levels a feature that is frequently observed in steatohepatitis (Table 1, p<0.05; n = 8–12). While treating dyslipidemic mice with VSL#3 had no effect on circulating levels of cholesterol, LDLc and HDLc, the probiotic effectively reduced AST and ALT plasma levels (Table 1, p<0.05; n = 8–12).

### VSL#3 improves insulin signaling in ApoE^–/–^ mice

**Figure 1:**
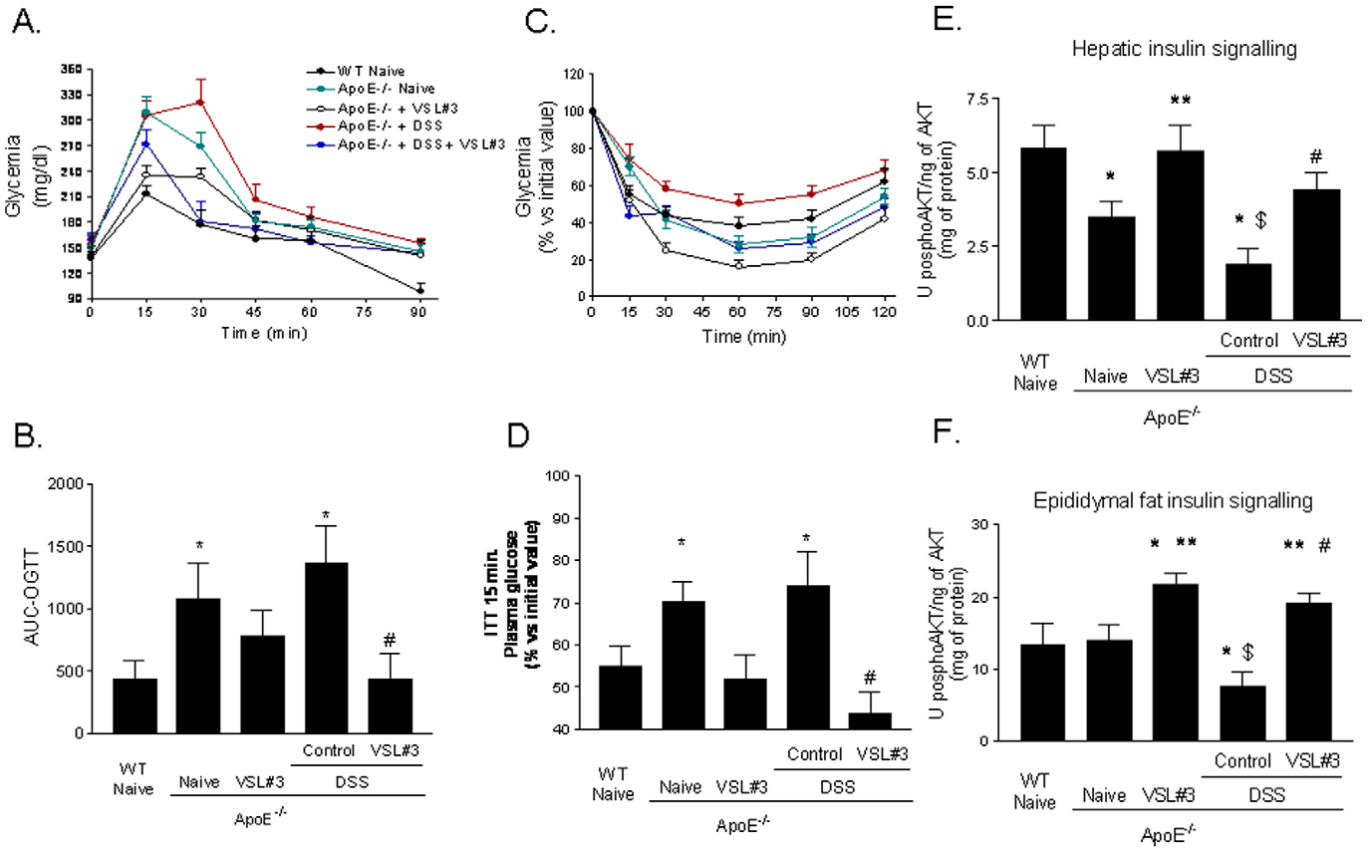
VSL#3 administration reverts insulin resistance in ApoE^−/−^ mice: effect on OGTT, ITT and insulin signaling. ApoE^−/−^ mice were administered daily with VSL#3 alone or in combination with DSS for 12 weeks starting at the age of 8–9 months. The OGTTs were performed after 10 weeks of treatment. **A**) Blood glucose levels in response to OGTT. **B**) Area under the OGTT curve (AUC) from 0 to 120 min after glucose administration: all curves were adjusted by subtracting the initial glucose values from actual glucose measurements. The ITTs were performed after 11 weeks of treatment, the data were expressed as % of basal glucose values . **C**) The ITTs were performed. **D**) illustrates glucose plasma levels after 15 minutes of insulin administration. Mean ± SE is plotted; n = 8–12 mice per group. Effect of VSL#3 administration on AKT Ser(473) phosphorylation in (**E**) liver and (**F**) epididymal fat tissues. Data are means ± SE of n = 8 mice per group. * p<0.05 ApoE^−/−^ experimental group versus naive wild type group; ** p<0.05 ApoE^−/−^ naïve group versus ApoE^−/−^ plus VSL#3 group; $ p<0.05 ApoE^−/−^ naive group versus ApoE^−/−^ plus DSS group; # p<0.05 ApoE^−/−^ plus DSS group versus ApoE^−/−^ plus DSS and VSL#3 group.

Confirming previous findings [29], after 8 hours of fasting, basal glucose plasma levels of naive ApoE^−/−^ mice were similar to that of wild type mice (Table 1), but insulin plasma levels were higher (Table 1, p<0.05; n = 8–12). Exposing ApoE^−/−^ to DSS failed to increase blood glucose levels, but increased insulin plasma levels from 7.8±0.8 to 9.3±0.7 μg/ml (Table 1; p = 0.14; n = 8–12). Treating ApoE^−/−^ mice with VSL#3 effectively reduced insulin levels to ∼5.5 μg/ml in both group (Table 1, p<0.05; n = 8–12). We have further characterized these mice by performing an OGTT and ITT in both wild type and ApoE^−/−^ naive mice and in ApoE^−/−^ mice exposed to DSS alone or in combination with VSL#3 for 10 or 11 weeks, respectively. As illustrated in Figure 1A there was a marked increase of glucose blood levels at 15 minutes after the oral load of glucose in naive ApoE^−/−^ mice compared to wild type naive mice (Figure 1 A and B; p<0.05; n = 8–12). Exposure of ApoE^−/−^ mice to DSS caused a further impairment of glucose tolerance (Figure 1 A and B, p<0.05; n = 8–12). VSL#3 administration improved OGTT tolerance in both naive ApoE^−/−^ mice and ApoE^−/−^ mice administered DSS (Figure 1; p<0.05; n = 8–12). The fact that ApoE^−/−^ mice were insulin resistant was confirmed further by results of the ITT. Thus, data shown in Figure 1C and D, demonstrate that ApoE^−/−^ mice were resistant to the insulin challenge (p<0.05; n = 8–12) and DSS treatment resulted in a further impairment of insulin activity (Figure 1 C and D, p<0.05; n = 8–12). The probiotic mixture restored the insulin signalling to levels compared to those of wild type mice (Figure 1 C and D, p<0.05; n = 8–12). As shown in Figure 1 E and F, ApoE^−/−^ old mice had an impaired, insulin signalling as measured by assessing the level of Serine threonine protein kinase (AKT) phosphorylation on ser-473 in the liver but not in the epididymal [30]. Interesting DSS-induced intestinal inflammation worsened insulin signalling in both tissues (Figure 1 E and F, p<0.05; N = 6). Treating naive or DSS-treated ApoE^−/−^ mice with VSL#3 increased the levels of AKT phosphorylation in both liver and epididymal fat (Figure 1 E and F, p<0.05; n = 8).

### VSL#3 intervention restored intestinal permeability and inflammatory markers

**Figure 2:**
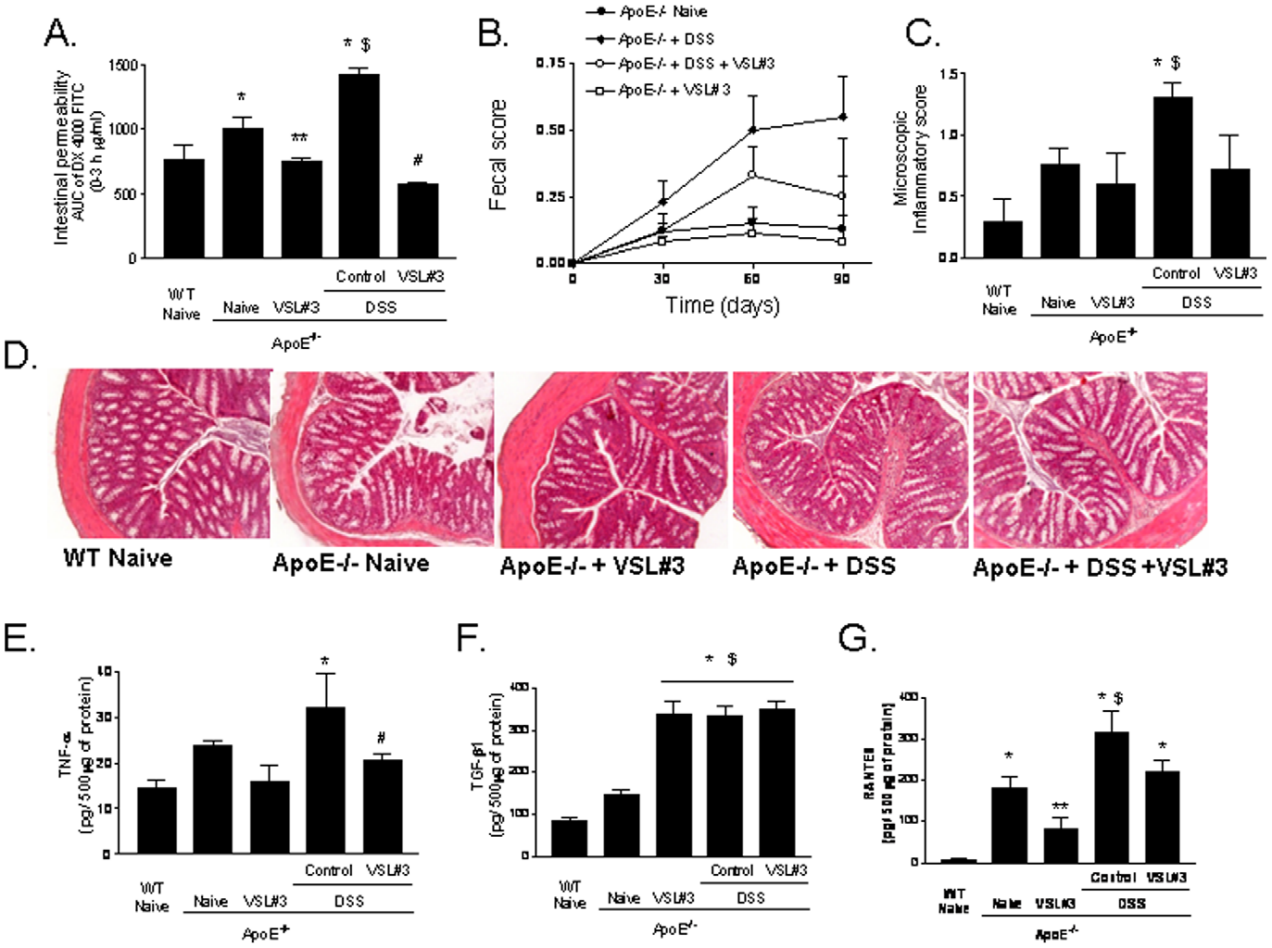
Effect of VSL#3 administration in intestinal homeostasis. A ) Intestinal permeability assay after oral challenge with 4000 Da fluorescent dextran-FITC (DX-4000-FITC); area under curve (AUC) of plasma DX-4000–FITC (μg/ml). **B**) Fecal score. **C**) Microscopy inflammatory score **D**) Histolopathology analysis of colon samples obtained from experimental groups. Original magnification 20×, H&E staining. Colonic cytokines content: (**E**) TNFα, (**F**) TGFβ1 and (**G**) RANTES. Data are mean ± SE of n = 7 mice per group. * p<0.05 ApoE^−/−^ experimental group versus naive wild type group; ** p<0.05 ApoE^−/−^ naïve group versus ApoE^−/−^ plus VSL#3 group; $ p<0.05 ApoE^−/−^ naive group versus ApoE^−/−^ plus DSS group;# p<0.05 ApoE^−/−^ plus DSS group versus ApoE^−/−^ plus DSS and VSL#3 group.

Compared to wild type naive mice, naive ApoE^−/−^ mice had a significant increase in intestinal permeability as measured by assessing plasma levels of DX-4000-FITC administered by an oral load (Figure 2 A, P<0.05; n = 7). Probiotics administration effectively improved epithelial barrier integrity in these mice [31], [32]. Furthermore, probiotics were effective in counteracting changes in intestinal permeability caused by DSS (Figure 2 A, P<0.05, n = 7). DSS treatment resulted in a subclinical inflammation and no changes in body weight and fecal score were recorded. However, histopathology assessment of the colon revealed a slight infiltration of colonic lamina propria by inflammatory cells (Figure 2 B and C). This finding was corroborated by the analysis of inflammatory mediators: indeed DSS challenge increased TNFα, TGF-β1 and RANTES content in the colonic mucosa (Figure 2 E–G, P<0.05; n = 7). VSL#3 intervention effectively counteracted these changes, attenuated the microscopic score and reduced mucosal levels of TNFα and RANTES (Figure 2 E and G; p<0.05; n = 7). No changes were measured in the mucosal levels of TGF-β1. Because TGF-β1 has potent anti-inflammatory activity, high concentrations of this cytokines in the colonic mucosa might support the anti-inflammatory activity of VSL#3 on inflammatory mediators.

### VSL#3 intervention reduces inflammation of mesenteric adipose tissues

**Figure 3:**
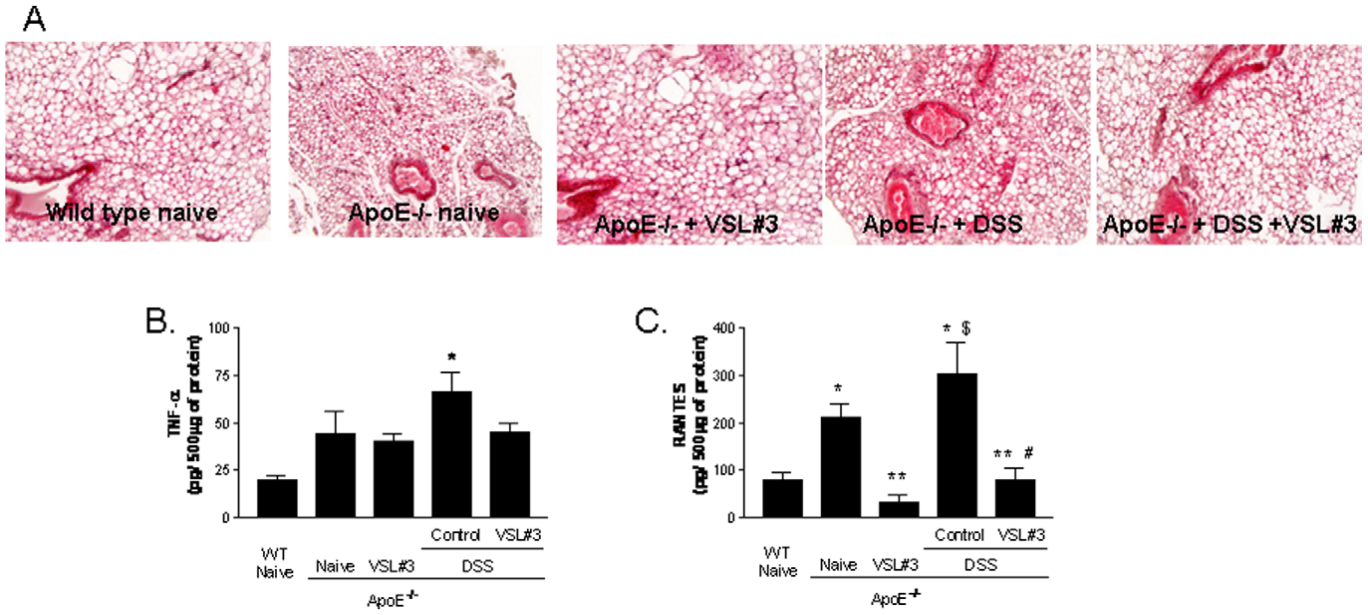
Effect of VSL#3 administration in mesenteric fat inflammation. A) Histopathology analysis of mesenteric fat isolated from experimental groups, original magnification 20×, H&E staining. Adipose tissue concentration of (**B**) TNFα and (**C**) RANTES. Data are mean ± SE of n = 5 mice per group. * p<0.05 ApoE^−/−^ experimental group versus naive wild type group; ** p<0.05 ApoE^−/−^ naïve group versus ApoE^−/−^ plus VSL#3 group; $ p<0.05 ApoE^−/−^ naive group versus ApoE^−/−^ plus DSS group; # p<0.05 ApoE^−/−^ plus DSS group versus ApoE^−/−^ plus DSS and VSL#3 group.

Adipose tissue is a major endocrine organ from which either metabolic and inflammatory signals propagate systemically [33], [34], exerting a wide range of regulatory functions beyond the intestinal wall. Compared to wild type naive mice the histophatology analysis of mesenteric fat isolated from ApoE^−/−^ mice revealed a moderate leucocytes accumulation in this tissue, associated with an alteration of adipocytes morphology (Figure 3 A) and increased levels of TNF-α and RANTES (Figure 3B). Exposure to DSS exacerbated the proinflammatory profile of mesenteric adipose tissue with a robust increase in the content of TNFα and RANTES (Figure 3 B and C; p<0.05 versus naive wild type; n = 5). These changes were reversed by VSL#3 intervention (Figure 3 A–C p<0.05; n = 5).

### VSL#3 intervention protects against development of liver inflammation and fibrosis

**Figure 4:**
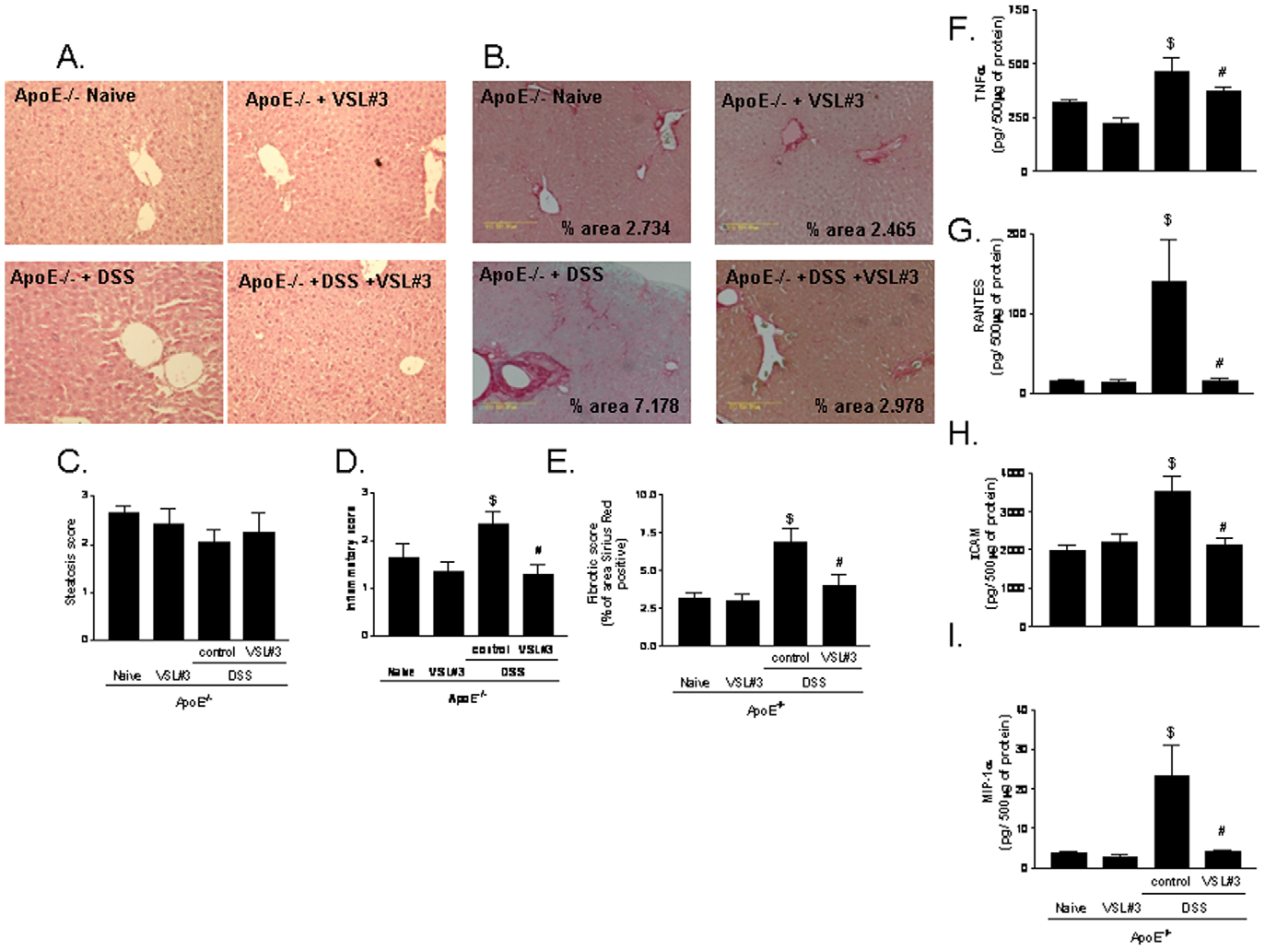
Effect of VSL#3 on liver inflammation and fibrosis induced by DSS administration. Histopathology analysis of liver, **A**) H&E staining and **B**) Sirus Red staining; original magnification 20×. **C**) Steatosis score; **D**) Inflammatory score and **E**) Fibrosis score. Data are mean ± SE of n = 8–12 mice per group. Liver content of inflammatory mediators including (**F**) TNFα, (**G**) RANTES, (**H**) ICAM-1, and (**I**) MIP-1α. Data are mean ± SE of n = 5 mice per group. * p<0.05 ApoE^−/−^ experimental group versus naive wild type group; ** p<0.05 ApoE^−/−^ naïve group versus ApoE^−/−^ plus VSL#3 group; $ p<0.05 ApoE^−/−^ naive group versus ApoE^−/−^ plus DSS group; # p<0.05 ApoE^−/−^ plus DSS group versus ApoE^−/−^ plus DSS and VSL#3 group.

Compared to wild type naive mice, naive ApoE^−/−^ mice had a higher steatosis score with no sign of inflammation (data not shown). Three months exposure of ApoE^−/−^ mice to DSS resulted in multiple changes of liver histopathology with the appearance major features of steatohepatitis. Thus, ApoE^−/−^ mice exposed to DSS developed a robust inflammation and fibrosis as indicated by the finding of multiple foci of inflammatory cells and by relatively intense Sirius Red staining of extracellular matrix proteins in hepatic sections (Figure 4 A–E; p<0.05; n = 8–12). These histopathology changes associated with increased levels of AST in the blood of ApoE^–/–^ mice challenged with DSS (Table 1). Further on, exposure of ApoE^−/−^ mice to DSS resulted in a robust increase in the liver content of inflammatory mediators including TNFα, RANTES, ICAM-1, and MIP-1α (Figure 4 F–I; p<0.05; n = 5). VSL#3 intervention failed to modulate the liver steatosis score in ApoE^−/−^ mice, but completely reverted the progression of liver inflammation and fibrosis in ApoE^−/−^ mice exposed to DSS (Figure 4 A–E; p<0.05; n = 8–12), and reduced the liver content of TNFα, RANTES, ICAM-1, and MIP-1α (Figure 4 F–I; p<0.05; n = 5).

### VSL#3 intervention protects against atherosclerosis development in ApoE^−/−^ mice

**Figure 5:**
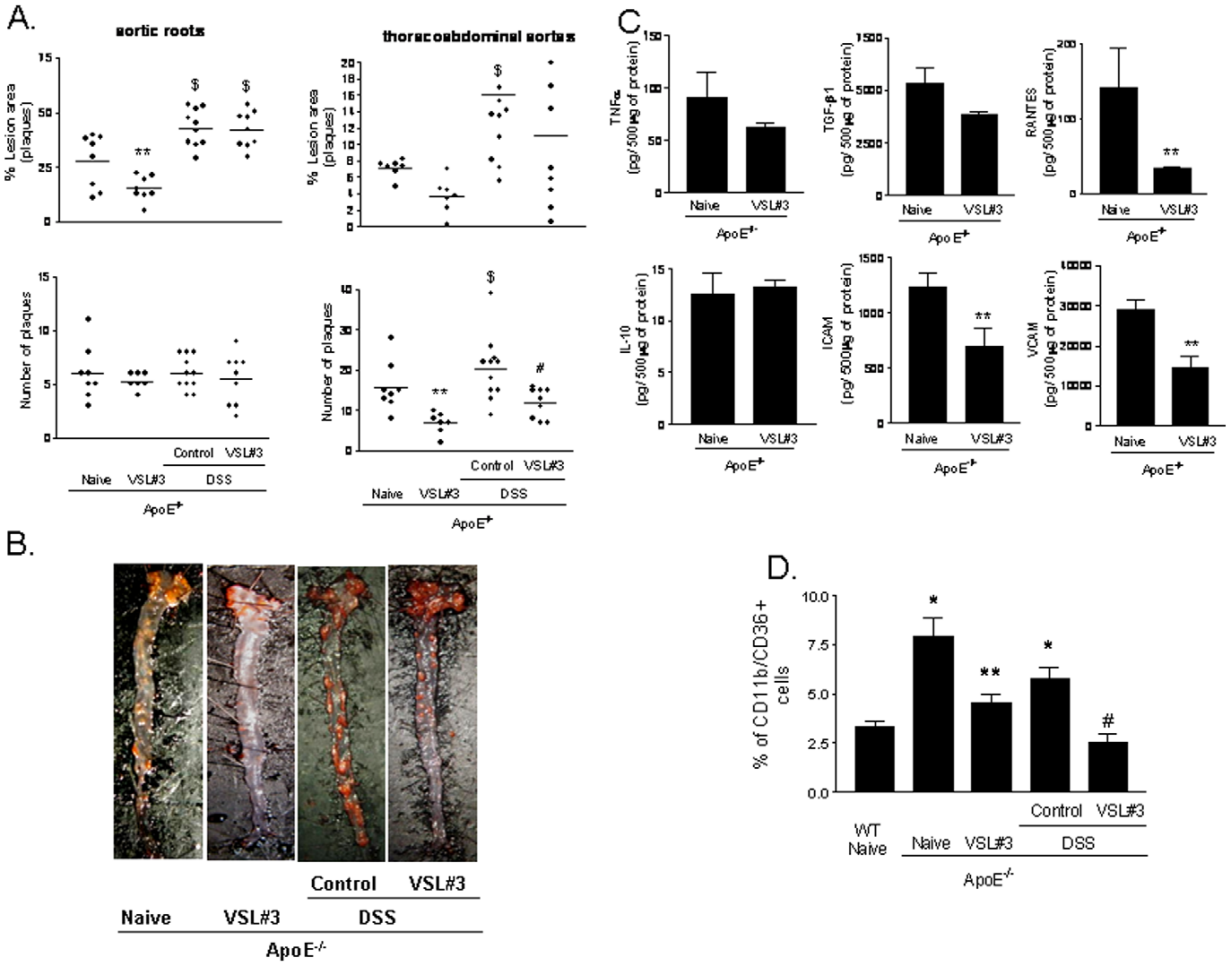
Effect of VSL#3 administration on atherosclerosis development in ApoE−/− mice. Panel A. Administration of DSS to ApoE^−/−^ for 12 weeks increases the size of aortic plaques (ratio of plaque surface area to vessel surface area) and numbers of plaques. Individual data are shown n = 7–12. **Panel B.** Representation of aortic plaques from individual animals. The images shows the plaque surface from individual animals, each one representative of a specific experimental group. The lipids in the vessel wall were staining with Sudan IV. **Panel C.** Aortic concentration of TNFα, TGF-β1, RANTES, IL-10, ICAM-1 and VCAM. Data are mean ± SE of n = 5 mice per group. **D**) VSL#3 intervention reduced the percentage of CD36 positive cells on circulating macrophages. The mean ± SE of n = 5 mice per group is shown. * p<0.05 ApoE^−/−^ experimental group versus naive wild type group; ** p<0.05 ApoE^−/−^ naïve group versus ApoE^−/−^ plus VSL#3 group; $ p<0.05 ApoE^−/−^ naive group versus ApoE^−/−^ plus DSS group; # p<0.05 ApoE^−/−^ plus DSS group versus ApoE^−/−^ plus DSS and VSL#3 group.

Analysis of Sudan IV staining of whole aortas, to measure neutral lipid content, demonstrated that ApoE^−/−^ mice at 12 months of age had severe atherosclerotic lesions: ≈26% of the aortic arch surface and ≈7% and thoracoabdominal tract surface. Administering mice with DSS exacerbated lipid accumulation extending the surface of atherosclerotic plaques up to ≈42% in the aortic arch and ≈15% in the thoracoabdominal aorta (Figure 5 A and B, p<0.05; n = 7–12). In addition, while the numbers of the plaques did not change among the experimental groups in the aortic arch, DSS treatment increased the number of the plaques in the thoracoabdominal aorta (Figure 5 A and B; p<0.05; n = 7–12), suggesting that exposure to DSS increases the formation of new plaques. This phenomenon was especially evident in the thoracoabdominal tract of aorta where the lipid accumulation in basal condition was minimal or moderate. Development of aortic plaque, in ApoE^−/−^ mice, was significantly attenuated by VSL#3 intervention (Figure 5 A, p<0.05; n = 7–8). Further on, administering DSS treated ApoE^−/−^ mice with VSL#3 prevented generation of new plaques in thoracoabdominal aortas and reverted the effect of DSS on the extension of atherosclerotic plaques (Figure 5A, p<0.05; n = 9–12). Additionally, the probiotics treatment effectively reduced the aortic levels of ICAM-1, VCAM and RANTES (p<0.05; n = 5). Furthermore, VSL#3 intervention effectively reduced the percentage of CD36 positive cells anong circulating macrophages as shown in Figure 5 D (P<0.05; n = 5).

### Treatment with VSL#3 induces IL-10-producing T-lymphocytes

**Figure 6:**
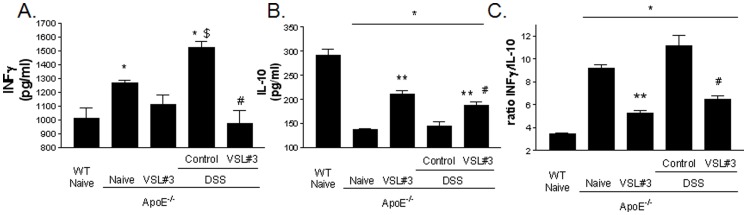
Effect of VSL#3 administration on the induction of T-lymphocytes IL-10-producing cells in spleen in ApoE^−/−^mice. T-lymphocytes were isolated from spleens of mice and then stimulated with concanavalin A for 36 h in vitro. INFγ and IL-10 in the supernatants were assayed by ELISA. The bar indicates mean ± SE of 5 samples from 5 mice in each group. * p<0.05 ApoE^−/−^ experimental group versus naive wild type group; ** p<0.05 ApoE^−/−^ naïve group versus ApoE^−/−^ plus VSL#3 group; $ p<0.05 ApoE^−/−^ naive group versus ApoE^−/−^ plus DSS group;# p<0.05 ApoE^−/−^ plus DSS group versus ApoE^−/−^ plus DSS and VSL#3 group.

Atherosclerosis is a chronic inflammatory disease involving activation of variety of immune cells including T lymphocytes, specifically CD4+ T cells with a T helper cell 1 (Th1) phenotype [35]–[37]. To investigate whether VSL#3 instructs the systemic immune system, CD5+ cells isolated from the spleen of different experimental groups were stimulated with concanavallin A for 36 h. Culture supernatants were examined for INFγ and IL-10 content. Compared to naive wild type mice, CD5+ cells isolated from naive ApoE−/− mice released higher amounts of IFNγ and 5-fold less IL-10 with a robust shift in the INFγ/IL-10 ratio, from 3.4 to 9.2 (Figure 6 A–C; p<0.05; n = 5). DSS administration caused a further shift toward a Th1 profile. This pattern was reversed by VSL#3 intervention in both naïve ApoE^−/−^ mice and ApoE^−/−^ mice challenged with DSS. Thus VSL#3 decreased INFγ release while caused a strong increase of IL-10 release (Figure 6A–C; p<0.05; N = 5). Similarly, spleen monocytes obtained from VSL#3 treated groups and stimulated with bacterial endotoxin in *vitro*, produced higher quantity of IL-10 compared to the others experimental groups (Figure S1; p<0.05; n = 5).

### VSL3 intervention modulates the expression of nuclear receptors in the intestine

**Figure 7:**
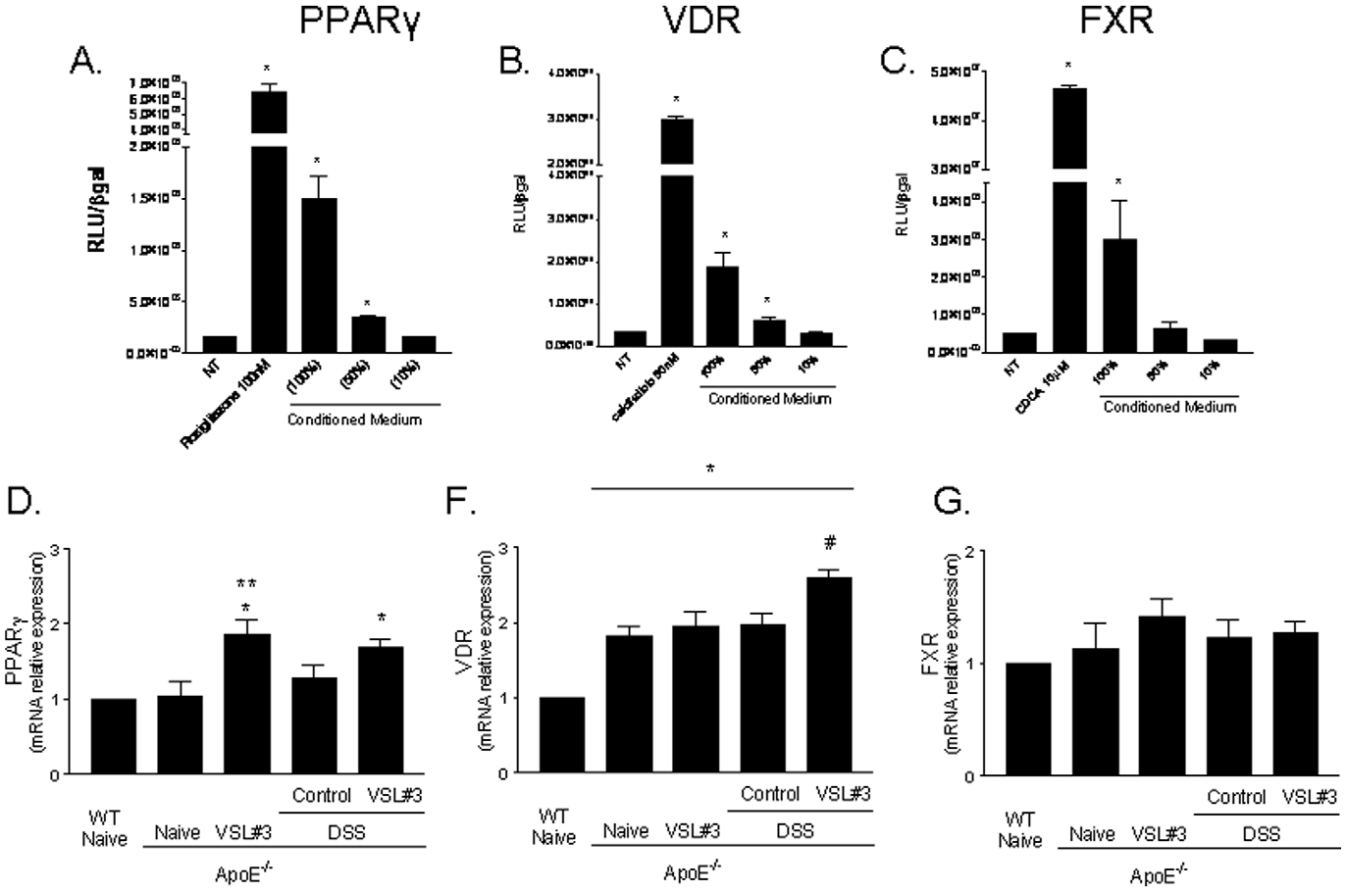
Effect of VSL#3 administration on PPARy, VDR and FXR, activity and expression. A ) Luciferase reporter assay performed in HepG2 transiently transfected with p(UAS)5-TKLUC, pSG5GAL4-PPARγ and pCMV-βgal vectors and stimulated 18 h with rosiglitazone 100 nM and condiditioned media (CM) undiluted, diluted 1∶2 (50%) and 1∶10 (10%). *p<0.05 *versus* not treated (NT); **B**) Luciferase reporter assay performed in HepG2 transiently transfected with p(UAS)5-TKLUC, pSG5GAL4-VDR and pCMV-βgal vectors and stimulated 18 h with 25-hydroxycholecalciferol, 50 nM, and CM undiluted, diluted 1∶2 (50%) or 1∶10 (10%). *p<0.05 *versus* not treated (NT); **C**) Luciferase reporter assay performed in HepG2 transiently transfected with p(hsp27)TKLUC, pSG5-FXR, pSG5-RXR, pCMV-βgal vectors and stimulated 18 h with CDCA 10 μM and CM undiluted, diluted 1∶2 (50%) and diluted 1∶10 (10%). *p<0.05 *versus* not treated (NT). (**Panel** **D–G**) RT-PCR analysis of the intestinal expression of PPARγ, VDR and FXR. Data represent the mean ± SE of 5 mice per group. * p<0.05 ApoE^−/−^ experimental group versus naive wild type group; ** p<0.05 ApoE^−/−^ naïve group versus ApoE^−/−^ plus VSL#3 group; $ p<0.05 ApoE^−/−^ naive group versus ApoE^−/−^ plus DSS group; # p<0.05 ApoE^−/−^ plus DSS group versus ApoE^−/−^ plus DSS and VSL#3 group.

Because probiotic intervention resets immunoactivation and metabolism in multiple organs, we have then investigated whether it modulate the expression of nuclear receptors involved in reciprocal regulation of immune system and metabolism. Previous studies have established a role for nuclear receptors in mediating the effects of probiotics in rodent models of inflammation [38]–[42]. Because an inverse regulation exists between several members of nuclear receptor superfamily and inflammation, we have assessed whether products of probiotic metabolism might directly regulate the activity of these regulatory factors. For this purpose, conditioned media obtained from VSL#3 cultures were tested for their ability to directly transactivate nuclear receptors and their relative agonistic activity was compared with that of canonical ligands, i.e. rosiglitazone for PPARγ, calcipherol for Vitamin D receptor (VDR) and chenodeoxycholic acid (CDCA) for FXR. HepG2 cells, an hepatocyte cell line expressing a large family nuclear receptors in a constitutive manner was used for this assay. While conditioned media failed to transactivate the glucocorticoid and the estrogen receptor (data not shown), they efficiently transactivated PPARγ, FXR and VDR (Figure 7 A–C). Because these data suggest a functional link between probiotic metabolism and nuclear receptors that are involved in regulating insulin sensitivity (PPARγ and FXR) and immune system (PPARγ, FXR and VDR), and probiotic metabolites could be absorbed by the intestinal epithelia and interact directly with the intestinal wall, we have then assessed the expression of these receptors in the intestine. Because As shown in Figure 7 D–E, expression of these receptors was modulated by probiotic intervention resulting in a significant increase in the intestinal expression of PPARγ and VDR in naive ApoE^−/−^ mice and mice challenged with DSS (p<0.05; N = 5).

## Discussion

The growing understanding of the functional role of human gut microbiota is showing that this enormous microbial population is instrumental in the control of host energy and lipid metabolism [43]–[47]. Thus, while metagenomic studies are progressively deciphering the role of bacterial genes and proteins in the regulation of host's metabolism, specific bacterial enterotypes have been associated to the development of human diseases such as diabetes and obesity [43]–[47]. Despite the relation of the intestinal microbiota with the host is mutual, the mechanisms by which the intestinal immune system copes with the gut microbiota to contain local inflammation and prevent systemic dysregulation of immunity and metabolism are still poorly defined.

In this report we have shown that low grade intestinal inflammation induced by administering ApoE^−/−^ mice with DSS results in a widespread inflammation whose signature markers were a systemic shift toward a Th1 phenotype along with a severe deterioration of the insulin signalling in the liver and adipose tissue. Because these changes were prevented by a probiotic intervention, these results highlight the central role of the intestinal microbiota in the pathogenesis of heretofore seemingly unrelated systemic inflammatory and metabolic disorders.

### Intestinal inflammation dysregulates systemic immunity and metabolism in ApoE^−/−^ mice exposed to DSS

The present study was designed to investigate whether intestinal inflammation could be a target in treating systemic disorders characterized by inflammation and altered insulin sensitivity. For this purpose we have worked out a model in which genetically dyslipidemic ApoE^−/−^ mice were fed DSS, an intestinal barrier breaking agent . ApoE^−/−^ mice *per se* develop biochemical features of insulin resistance, lipid abnormalities and liver steatosis but fail to develop NASH like features unless they are feed an high fat diet. Results of our studies demonstrate that development of NASH-like lesions in the liver in ApoE^−/−^ mice fed DSS does not require an high fat diet, strongly advocating a regulatory role of the intestinal microbiota on systemic metabolism. Development of NASH like features in this model was supported by a robust increase of intestinal permeability, a marker of intestinal epithelial barrier dysfunction, that would provide a low grade endotoxinemia and systemic immune activation. Thus, feeding ApoE^−/−^ mice with DSS provides a “*second hit*” that suffices to promote transition from NAFLD to NASH [22]–[26]. Because these changes were not supported by any additional modification of lipid metabolism (Table 1), and were prevented by a probiotic intervention, this model could be exploited to disentail the pathogenic role of intestinal microbiota in systemic disorders.

Of interest, naive ApoE^−/−^ mice were characterized by an altered intestinal permeability along with a systemic polarization of immune system toward a Th1 phenotype [48], [49]. ApoE^−/−^ mice develop a low grade of intestinal inflammation that manifests it self with increased accumulation of inflammatory cells in the intestinal wall along with an increased expression of markers of inflammation including TNFα and RANTES. Despite the mechanisms that drive intestinal inflammation in ApoE^−/−^ mice are poorly defined, the ApoE protein itself counter-regulates TLR-3 and 4 signalling [49] and its deficiency results in a shift toward a Th1 phenotype. In short, present results highlight a previously undetected role for ApoE in the regulation of intestinal homeostasis [49].

### Role of probiotics intervention in regulating intestinal inflammation and immune and metabolic dysfunction in ApoE^−/−^ mice exposed to DSS

Exacerbation of systemic inflammation and deterioration of insulin signalling caused by DSS administration to ApoE^−/−^ mice was reversed by modification of intestinal microbiota with VSL#3. Thus, while VSL#3 treatment had no effect on plasmatic lipid profile, it effectively attenuated intestinal inflammation and ephitelial barrier dysfunction. VSL#3 administration sufficed to reverse the shift toward a Th1 phenotype caused by DSS and improved insulin signalling in the mesenteric fat and liver. These biochemical changes associated with a significant improvement of liver and aortic pathology. The mechanism(s) by which VSL#3 intervention attenuates immune dysfunction and resets insulin signalling are multiple, and certainly include attenuation of intestinal epithelial barrier dysfunction. However, we have also provided evidence that metabolites of VSL#3 probiotics regulate the expression/function of nuclear receptors involved in a multilevel regulation of immunity and metabolism (see below).

VSL#3 has been demonstrated to be effective in other models of NASH (50,51). Thus, VSL#3 intervention ameliorates liver fibrosis in the methionine-choline-deficient diet-induced mouse model of NASH [50] while attenuates liver steatosis in *ob/ob* mice feed an high fat diet [51]. In former model VSL#3 failed to protect against development of liver steatosis or inflammation but ameliorated the liver fibrosis resulting in diminished accumulation of collagen and α-smooth muscle actin, i.e. two markers of fibrosis [50]. Of interest these effects were associated with an increased liver expression of PPARs and impaired TGFβ signaling [50]. While these preclinical studies are promising, there are only small clinical trails carried out in NASH patients. These studies have shown some beneficial effects of VSL#3 treatment highlighting the need for controlled clinical trials [52].

The VSL#3 preparation is a mixture of eight different probiotics, thus leaving the question of which specific probiotic(s) might be responsible for the therapeutic improvement described in this study. Unfortunately, there are no data on the effects of specific probiotic in preclinical models of NASH or NASH patients.

### Vascular effects of probiotics in ApoE−/− mice administrated with DSS

Present results show that transition from NAFLD to NASH in ApoE−/− mice feed DSS associates with a deterioration of insulin signalling. This observation fit with the notion that NASH is the hepatic component of the metabolic (insulin resistance) syndrome [8]–[10]. Because insulin-resistance associates with increased morbidity and mortality from cardiovascular diseases, the present model might offer insights on the role exerted by intestinal inflammation on development of insulin resistance and atherosclerosis [8]–[10]. NASH associates with increased risk of cardiovascular disorders. Surrogate markers of atherosclerosis (e.g., carotid intima-media thickness) are highly prevalent in NAFLD, and clinical endpoints such as deaths from myocardial infarction/need for coronary revascularisation have been documented in several natural history studies of NAFLD [8]–[10]. There is some evidence that individuals with NASH have a worse atherogenic profile, and are more likely to have overt cardiovascular disease than patients with hepatic steatosis alone [53], [54].

Here, we have shown that intestinal inflammation might be a co-factor for development of severe atherosclerotic lesions in a model of genetic dyslipidemia and NASH. The finding that VSL#3 protects against aortic plaques formation suggests that the intestinal microbiota could be target for treating cardiovascular complication associated with NASH.

### Probiotics intervention modulates the expression/activity of nuclear receptors in the intestine and mesenteric fat

Results from the present study suggest that the shift toward a more tolerogenic immune-phenotype caused by VSL#3 intervention, could be mediated by the regulation of the expression/activity of members of nuclear receptor superfamily [55]–[59]. Indeed, products of probiotics metabolism directly transactivate PPARγ, FXR and VDR *in vitro* and increase expression of these regulatory factors, PPARγ and VDR, *in vivo*. Since these nuclear receptors exert their regulatory function at the interface between glucose and lipid metabolism and immune system [55]–[59], and their colonic expression is downregulated in rodent models of colitis [39], as well as in patients with inflammatory bowel diseases [56], their modulation might be instrumental to the effects exerted by VSL#3.

Previous studies have shown that commensal anaerobic gut bacteria attenuate intestinal inflammation by counter-regulating NF-κB activity by a PPAR-γ mediated mechanism [39]. The molecular mechanism that support this regulatory effect on PPARγ are only partially resolved, but evidence is growing that bacterial metabolites could be involved. Indeed, recent studies have shown that VSL#3 intervention modulates lipid content and xenobiotic signaling pathway by increasing the intestinal content of conjugated linoleic acid (CLA), a positional and geometric isomers of linoleic acid and a known agonist for PPARγ [41], [42], [57]. Interestingly, while VSL#3 was found to directly generate CLA [42] and CLA content increases in the gut of VSL#3-treated mice, CLA generated under VSL#3 treatment is not absorbed or distributed systemically, suggesting that products of probiotic metabolism act locally to regulate nuclear receptor expression/function [42]. Because PPARγ is a potent insulin sensitizer and its activation in macrophages downregulates the expression of a number of inflammatory mediators [60], its trans-activation might contribute to the spectrum of beneficial effects exerted by VSL#3 in this model. Of interest, VSL#3 increases the liver expression of PPARγ in mice feed a choline deficient diet.

Probiotic products also transactivate FXR. FXR is a bile acid sensor regulated by relative concentrations of primary bile acids [56], [58]. In the intestine and liver activation of FXR by its natural ligand, chenodeoxycholic acid (CDCA), regulates a number of genes involved in bile acids uptake, conjugation and excretion [56], [58]. Additionally, mice that are deficient for FXR develop spontaneous inflammation and insulin resistance [58] with age, while FXR ligands have been demonstrated beneficial in reducing atherosclerotic plaque formation and intestinal inflammation [59], [61]–[63]. Furthermore, activation of FXR in the intestine releases fibroblast growth factor (FGF)15, a potent insulin sensitizer [64]. Interesting, sodium butyrate, a well characterized product of VSL#3 metabolism induces FGF21 expression in the liver [65]. Because activation of FXR is a defined target in treating metabolic syndrome and specific ligands for this receptor are tested in NASH patients [66] our data suggest, that similarly to PPARγ, activation of FXR might contribute to the beneficial effects observed under VSL#3 intervention.

Finally, we have shown that VSL#3 transactivates the VDR. Because VDR regulates intestinal immune system and VDR ligands attenuates intestinal and systemic inflammation [67], its activation might also support anti-inflammatory and immunomodulatory effects exerts by VSL#3 in the present study.

In summary, we have shown that intestinal inflammation is a driving factor for development of steatohepatitis and atherosclerosis in a genetic model of dyslipidemia. VSL#3 intervention corrects inflammation-driven insulin resistance and protects against development of NASH and atherosclerosis in a rodent model of genetic dyslipidemia and intestinal inflammation.

## Materials and Methods

### Animal and treatments

C57BL6 male mice were from the Harlan Nossan (Udine, Italy). ApoE^–/–^ male mice (supplied by the animal centre of the University of Perugia) were housed under pathogen-free conditions. Mice were housed under controlled temperatures (22°C) and photoperiods (12∶12-hour light/dark cycle), allowed unrestricted access to standard mouse chow and tap water. Protocols were approved by the University of Perugia Animal Care Committee according to the Italian guideline for care and use of laboratory animals. The ID for this project is #98/2010-B. The authorization was released to Prof. Stefano Fiorucci, as a principal investigator, on May 19, 2010. Eight to nine months old ApoE^–/–^ mice were randomized into four groups (N = 13) : group 1, saline orally (150 μl/mouse/day); group 2 probiotics, orally, at dose of 20×10^9^ colony-forming units (cfu)/kg/day (25×10^8^/mouse); groups 3 and 4 received Dextran sulfate sodium (DSS) (molecular weight, 40 kDa; ICN Biomedicals Inc.,) 0.2% in filtered drinking water plus saline orally (150 μl/mouse/day) or probiotics, orally, at dose of 20×10^9^ colony-forming units (cfu)/kg/day (25×10^8^/mouse), respectively. Probiotics were administered six days a week for 12 weeks. The mice were monitored weekly for weigh, fecal score and anal bleeding and food intake. At the end of the experiment animals were sacrificed and blood collected for subsequent biochemical assays and flow cytometry analysis while tissues were snap frozen for RNA and protein isolation and histology. The aortas were processed for enface atherosclerotic lesion coloration and spleens for lymphocytes isolation. Serum content of total cholesterol, triglyceride, HDL, LDL, AST and ALT were measured by enzymatic assays (Wako Chemicals; Osaka, Japan). Serine threonine protein kinase (AKT) phosphorylation on Ser(473) were assessed in liver, and epididymal fat adipose samples using a validated, total Akt Elisa kit, Akt p-S473 Elisa kit from BIOSOURCE (Invitrogen, Milan, Italy).

### Reagents

All reagents were purchased from Sigma Aldrich (Milan, Italy). The probiotics compound VSL#3, consisting of 8 strains of bacteria (L. acidophilus MB 443, L. delbrueckii subsp. bulgaricus MB 453, L. casei MB 451, L. plantarum MB 452, B. longum Y10, B. infantis Y1, B. breve Y8, and S. salivarius subsp. thermophilus MB 455), was from VSL Pharmaceuticals.

### Quantification of atherosclerotic plaques

Aortas (N = 7–13 for group) were dissected for quantification of atherosclerosis as previously described [58].

### Liver histopathology

For histological examination, portions of the right and left liver lobes (10–15 mg/each) from each animal were fixed in 10% formalin, embedded in paraffin, sectioned, and stained with haematoxylin and eosin or Sirius red. For the latter, sections were incubated for 30 minutes in 0.1% Sirius red F3B (Sigma Chemical Co.) containing saturated picric acid and 0.1% fast green. After rinsing twice with distilled water, sections were briefly dehydrated with 70% ethanol. Collagen surface density from liver samples was quantified using a computerized image. Images were acquired with a BX60 microscope (Olympus Co., Rome, Italy) and a digital camera (Digital Microscope Camera ProgResC14, Jenoptik, Germany) and analyzed by specific software (Delta Sistemi, Rome, Italy) with a resolution of 1315×1033 pixels. The surface density of collagen in blinded specimens was measured at a video screen display magnification and expressed as a percentage (the ratio of collagen surface area per total analyzed field surface). All the hepatic compartments were analyzed at low magnification. The average of the score taken from 10 random fields was used to generate a single score for each liver. Liver histological sections (4 µm) were prepared and stained with H&E. For steatosis and inflammatory scores, 3 sections from each mice were graded blindly. A score of 0 to 3 was used to describe the extent of steatosis. A zero score represents normal liver structure in which lipid accumulation in the hepatocytes is not observed (<5%). A score of 1 depicts mild lipid infiltration (occurring in, 5–33%; hepatocytes), a score of 2 depicts moderate lipid infiltration (occurring in >33–66%; hepatocytes), whereas a score of 3 indicates severe lipid infiltration (occurring in >66%.of hepatocytes). The same sections were analyzed for inflammatory score (from 0 to 3) (0 =  No foci/200x; 1 = <2 foci/200x; 2 = 2–4 foci/200x; 3 = >4 foci/200x.).

### Colon histopathology

Sections (7 mice per group) from the proximal colon were fixed in buffered formalin, and routine 5-μm sections were prepared and stained with hematoxylin and eosin. Stained sections were examined blindly and scored for the extent of inflammation, using the following score: 0 =  none; 1 =  mucosal; 2 =  mucosal + submucosal involvement; 3 =  mucosal + submucosal + muscle penetrate; 4 =  full thickness involvement.

### Oral glucose tolerance test (OGTT)

Glucose tolerance test was performed on the 10^th^ week of the study after an overnight fasting of 8 hours (N = 6 per group). Mice were given 2 g/kg glucose orally, and blood samples were collected at 0, 20, 40, 60, 100, 120 minutes after glucose loading via the tail vein and glucose levels measured by blood glucose meter. The basal plasmatic levels of insulin was measured by ELISA kit (Mercodia). The linear trapezoidal method uses linear interpolation between data points to calculate the AUC.

### Insulin tolerance test (ITT)

ITT was performed on the 11^th^ week of the study (N = 6 per group). Mice were injected intraperitoneally with human insulin, 4 U/kg, (Sigma) and blood samples collected via the tail vein at 0, 20, 40, 60,100 and 120 minutes after insulin administration and glucose levels measured by blood glucose meter. The data were expressed as % versus the value of blood glucose at time 0.

### Intestinal permeability assay

This measure is based on the intestinal permeability towards 4000 Da fluorescent dextran-FITC (DX-4000-FITC) (FD4000; Sigma-Aldrich, St. Louis, Missouri, USA). The test was performed 3 weeks before the end of the study. Briefly, mice that were fasted for 6 h were given DX-4000-FITC by gavage (500 mg/kg body weight, 125 mg/ml). Blood samples were collected 1 and 3 hours after dextran-FICT administration and centrifuged at 4°C, 12.000 g for 3 minutes. Plasma were diluted in an equal volume of PBS and analysed for DX-4000-FITC concentration with a fluorescence spectrophotometer at an excitation wavelength of 485 nm and emission wavelength of 535 nm. A standard curve was obtained by diluting FITC-dextrane in non-treated plasma diluted with PBS (1∶3 v/v). The linear trapezoidal method uses linear interpolation between data points to calculate the AUC.

### Isolation and culture of spleen monocytes and lymphocytes

Mouse immune cells were obtained from spleens (N = 4 for group). Monocytes were isolated by positive selection using magnetic cell sorting according to the manufacturer's instructions (Miltenyi Biotec). Therefore the remaining cells were used to isolate T-lymphocytes by CD5 microbeads according to the manufacturer's instructions (Miltenyi Biotec). After isolation, monocytes were resuspended in complete RPMI medium at the concentration 1×10^6^/ml and T-lymphocytes at 2×10^6^/ml and cultured in a 24 well plate for 36 hours alone or in combination of LPS (2 μg/ml) and ConcanavallinA (2 μg/ml), respectively. At the end of incubation the supernatants were collected cytokines assay by ELISA kit (SaBioscence).

### Transactivation experiments

HepG2 cells were plated in a 12-well plate at 2·10^5^ cells/well in “Minimum Essential Medium with Earle's salts” (E-MEM) containing 10% fetal bovine serum (FBS), 1% L-glutamine and 1% penicillin/streptomycin. The transfection experiments were performed using Fugene HD (Roche) according to manufactured specification. For PPARγ and VDR mediated transactivation, cells were transfected with 500 ng reporter vector p(UAS)_5X_TKLuc, 200 ng pCMV-βgalactosidase and with 300 ng of a vector containing the ligand binding domain of PPARγ or VDR, respectively, cloned upstream of the GAL4-DNA binding domain. For FXR mediated transactivation, cells were transfected with 150 ng pSG5-FXR, 150 ng pSG5-RXR, 200 ng pCMV-βgalactosidase and with 500 ng of the reporter vector p(hsp27)-TK-LUC containing the FXR response element IR1 cloned from the promoter of heat shock protein 27 (hsp27). At 24 h post-transfection, cells were starved using E-MEM (cell culture medium) free of serum and supplemented with 1% L-glutamine and 1% penicillin/streptomycin and stimulated 18 h with the appropriate nuclear receptor agonist. To evaluate if VSL#3 probiotics mixture produced molecules with transactivation capacity on FXR, PPARγ and VDR, HepG2 were stimulated with a conditioned medium (CM). To prepare CM, 10 mg of VSL#3 probiotics formula was reconstituted in 10 ml of serum/antibiotic-free E-MEM cell culture medium and was grown overnight in medium at 37°C without shaking. The CM was centrifuged at 4,100 rpm for 10 min to separate the bacteria, and the resulting supernatant was filtered two times through a 0.22- µm membrane (Millipore) to remove any insoluble particles and supplemented with 100 U/mL penicillin-streptomycin and 2 mM L-glutamine. The pH of the buffer was adjusted to 7.4 and then filtered through a 0.22- µm filter. HepG2 were stimulated with CM undiluted, diluted 1∶2 and 1∶10 with E-MEM. After treatments, cells were lysed in 100 µl lysis buffer (25 mM TRIS-phosphate pH 7,8; 2 mM DTT; 10% glycerol; 1% Triton ×100) and 10 µl cellular lysate was assayed for luciferase activity using the Luciferase Assay System (Promega). Luminescence was measured using an automated luminometer. Luciferase activities were normalized for transfection efficiencies by dividing the relative light units by β-galactosidase activity expressed from cells cotransfected with pCMV-βgal.

### Real-Time PCR

Quantization of the expression level of selected genes was performed by quantitative real-time PCR (qRT-PCR). Total RNA were obtained from intestinal pieces (100–50 mg) and isolated with TRIzol reagent (Invitrogen, Milan, Italy), incubated with DNase I and reverse-transcribed with Superscript II (Invitrogen) according to manufacturer specifications. For real-time PCR, 25 ng of template was used in a 25-µl reaction containing a 0.3 µM concentration of each primer and 12.5 µl of 2x SYBR Green PCR Master Mix (Bio-Rad Laboratories, Hercules, CA). All reactions were performed in triplicate using the following cycling conditions: 2 min at 95°C, followed by 50 cycles of 95°C for 10 s, 58–60°C for 30 s and 72°C for 30 s using an iCycler iQ instrument (Bio-Rad Laboratories). The mean value of the replicates for each sample was calculated and expressed as cycle threshold (CT). The amount of gene expression was then calculated as the difference (ΔCT) between the CT value of the sample for the target gene and the mean CT value of that sample for the endogenous control (GAPDH). Relative expression was calculated as the difference (ΔΔCT) between the ΔCT values of the test and control samples for each target gene. The relative level of expression was measured as 2-ΔΔCT. All PCR primers were designed using the software PRIMER3-OUTPUT using published sequence data obtained from the NCBI database.

Mouse primers were as follows:

GAPDH: ctgagtatgtcgtggagtctac and gttggtggtgcaggatgcattg.

FXR: tgtgagggctgcaaaggttt and acatccccatctctctgcac.

PPARγ: gccagtttcgatccgtagaa and aatccttggccctctgagat.

VDR: tcacagatgaggaggtgcag and gagcaggatggcgataatgt.

### Statistical Analysis

All values are expressed as mean ± SE of “n” experiments. The statistical analysis was carried out by GraphPad Prism software. Comparisons of more than 2 groups were made with a 1-way analysis of variance with post hoc Tukey tests. Differences were considered statistically significant if P was <0.05.

## Supporting Information

Figure S1 **Administration of VSL#3 induces monocytes/macrophages IL-10-producing cells in spleen in ApoE−/− mice.** Monocytes/macrophages were isolated from spleen of the mice of experimental group stimulated with LPS for 36 h in vitro. INFγ and IL-10 in the supernatants were assayed by ELISA. The bar indicates mean ± SE of 5 samples from 5 mice in each group. * p<0.05 ApoE^−/−^ experimental group versus naive wild type group; ** p<0.05 ApoE^−/−^ naïve group versus ApoE^−/−^plus VSL#3 group; $ p<0.05 ApoE^−/−^ naive group versus ApoE^−/−^plus DSS group; # p<0.05 ApoE^−/−^ plus DSS group versus ApoE^−/−^ plus DSS and VSL#3 group.
(TIF)
