# Supplementary figures and images for: VSL#3 Resets Insulin Signaling and Protects against NASH and Atherosclerosis in a Model of Genetic Dyslipidemia and Intestinal Inflammation
Source: PLoS One. 2012 Sep 21;7(9):e45425. doi: 10.1371/journal.pone.0045425 (PMC3448636; doi:10.1371/journal.pone.0045425)

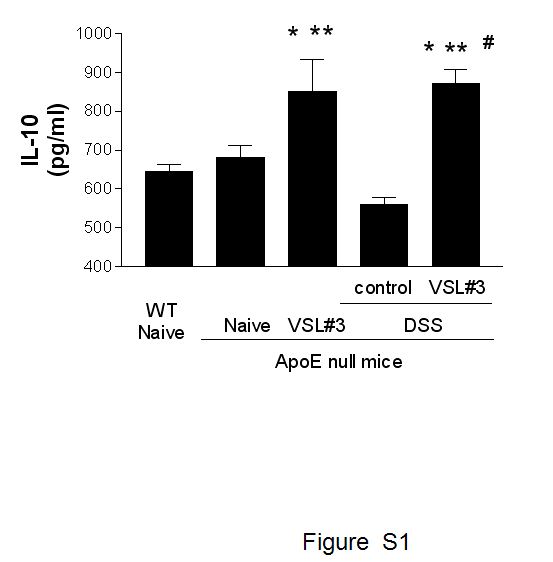

Supplement: Figure S1 — Administration of VSL#3 induces monocytes/macrophages IL-10-producing cells in spleen in ApoE−/− mice. Monocytes/macrophages were isolated from spleen of the mice of experimental group stimulated with LPS for 36 h in vitro. INFγ and IL-10 in the supernatants were assayed by ELISA. The bar indicates mean ± SE of 5 samples from 5 mice in each group. * p<0.05 ApoE−/− experimental group versus naive wild type group; ** p<0.05 ApoE−/− naïve group versus ApoE−/−plus VSL#3 group; $ p<0.05 ApoE−/− naive group versus ApoE−/−plus DSS group; # p<0.05 ApoE−/− plus DSS group versus ApoE−/− plus DSS and VSL#3 group. (TIF) [file pone.0045425.s001.tif]
